# Determinants of self-perceived health for Canadians aged 40 and older and policy implications

**DOI:** 10.1186/s12939-017-0595-x

**Published:** 2017-06-06

**Authors:** William Ian Andrew Bonner, Robert Weiler, Rotimi Orisatoki, Xinya Lu, Mustafa Andkhoie, Dana Ramsay, Mohsen Yaghoubi, Megan Steeves, Michael Szafron, Marwa Farag

**Affiliations:** School of Public Health, University of Saskatchewan, 104 Clinic Place, Room 3334, Saskatoon, SK S7N 5E3 Canada

**Keywords:** Self-perceived health, Chronic disease, Stress, Social determinants of health, Older adults, Healthy public policies

## Abstract

**Background:**

Perceived health status indicates people’s overall perception of their health, including both physical and psychological dimensions. The aim of this study was to examine the determinants of self-perceived health for Canadians aged 40 and older using data from the Canadian Community Health Survey (2010).

**Methods:**

Multiple logistic regression models were employed to identify factors associated with self-perceived health in two age groups: Adults aged 65+ and Adults aged 40–64.

**Results:**

We found that higher income was significantly associated with better health status while chronic conditions and stress were associated with worse health status. In the 40–64 and 65+ age groups, individuals in the highest income bracket were 4.65 and 1.94 times, respectively, more likely to report better health than individuals in the lowest income bracket. The difference in the level of income associated health inequities between the two age groups point to the need for understanding the reasons behind lower inequities among seniors and how much the social protections provided by the Canadian government to seniors contribute to lowering inequities.

**Conclusions:**

Though Canada has a national public health insurance system providing coverage to all Canadians, health inequities associated with income persist providing further evidence of the importance of the social determinants of health. Examining the extent of these inequities and what factors influence them helps direct policy attention. In addition to documenting inequities, this paper discusses policy options for reducing the identified inequities.

## Background

There is an urgent need for health systems to have timely information on the needs and demands of the population accessing its services. Evidence from existing studies supports the use of individual ratings [1–3] to approximate *self-determined perception of health status* [4, 5]. Self-perceived health status is a predictor of morbidity and mortality [6–11], physical functioning [12, 13], and utilization of health services [14–16]. Furthermore, self-perceived health status ratings are highly correlated to physician assessments of health conditions [17, 18]. Finally, it is very important to assess the extent to which self-perceived health inequities exist and what drives them.

The Canadian government has collected survey data on self-perceived health and associated outcomes and health service utilization patterns for several decades. A 2001 Health Report prepared by Statistics Canada demonstrated that both social and psychological factors are predictors of self-perceived health status [19]. Improved understanding of the determinants of self-perceived health could be used to inform health promotion activities.

More recently, attention has been paid to how self-perceived health changes over time and varies across age groups. Such insights are increasingly important in light of the demographic changes associated with the maturation of Canada’s “baby boom” [20, 21]. As perceived health status impacts the demand for health services, access to this information will permit planners to better meet the changing needs of an aging population [22, 23]. We also examined the factors associated with self-perceived health to identify the extent to which inequities exist and what drives them. To address inequities, we need to clearly understand what factors contribute to health inequities in the Canadian context and by how much.

## Methods

This study used data from the 2010 Canadian Community Health Survey (CCHS). For more information regarding the survey methodology, please refer to the website provided [24]. Survey weights, provided by Statistics Canada were used to account for oversampling and under sampling as described by Statistics Canada [24, 25]. Stata version 12.1 was used to conduct all analyses presented in this study. The level of significance was set at 0.05.

Our outcome of interest was dichotomized self-perceived health categorized as following: Good health (1) if respondents answered excellent, very good or good to the following question “In general would you say your health is?” and Less than good health (0) if respondents answered fair or poor to the same question. In this way, we identified those conditions associated with less than “*good*” perceived health. The study variables were selected from the 2010 CCHS based on the results of previous studies that identified major determinants of perceived health status [1–4]. The selected factors were grouped into three broad categories according to the format denoted in the 2010 CCHS, and include (a) social determinants, (b) psychological determinants and (c) physiological conditions in addition to control variables (age and sex).

Inclusion of social determinants as model variables was used to estimate the association of self-perceived health with social factors, including: level of education (less than secondary, secondary grad, other post secondary and post secondary grad); household income (<$20,000, $20,000–$39,999, $40,000–$59,999, $60,000–$79,999 and $80,000 or more); and marital status (married, living common-law, widowed, separated, divorced or single/never married). To assess the relationship between self-perceived health and mental health factors, perceived stress was included as a model variable under the psychological determinants category. According to the CCHS, perceived stress was defined by respondents as the amount of stress they experience most days (not at all stressful, not very stressful, a bit stressful, quite a bit stressful or extremely stressful). Lastly, physiological conditions were included based on CCHS’s classification of chronic conditions. Conditions classified in this way include asthma, arthritis, hypertension, diabetes, heart disease, cancer and anxiety disorder, to which respondents indicated having the condition or not.

We used a purposeful model selection strategy to fit the final logistic regression models. First, univariate analysis was conducted for each independent variable. Additionally, a standard contingency table was created for each categorical variable to determine if the cell frequency was equal to zero for either of the model’s outcomes (i.e. “*good health”* or “*poor health*”). Variables were selected for inclusion if they contributed to the model (i.e., with a *P*-value less than 0.20). Based on the results of the univariate analyses, marital status was excluded because it did not contribute to the model for either the 40–64 age group (0.89≥0.25) or the 65 and older age group (0.73≥0.25). Full results of the univariate analyses are shown in Table 1. Second, two multivariate regression analysis models were used: (1) multiple logistic regression model nested within age group 65 and older and (2) multiple logistic regression model nested within age group 40–64. In model 1 above, 12,044 observations were used (23.5% missing) in the final model. In model 2 above, 20,365 observations were used (15.0% missing) in the final model. We performed the Hosmer-Lemeshow test to assess the goodness of fit of the logistic regression models; the *p*-values for our models were 0.35 and 0.43 for the 40–64 years model and 65 above model respectively, which indicates that the two models fit the data well. In addition, we used the Linktest to examine model specification; the *p*-values for the _hatsq were 0.24 for the 40–46 years model and 0.36 for the 65 above model, which means that there is no specification error.

**Table 1:** Descriptive and univariate analyses of self-perceived health and explanatory variables

Characteristics	Total (%)	GOOD HEALTH=1 *N* (%)	LESS THAN GOOD HEALTH=0 *N* (%)	UNI OR	*P* value	Total (%)	GOOD HEALTH=1 *N* (%)	LESS THAN GOOD HEALTH=0 *N* (%)	UNI OR	*P* value
	AGE 40–64					AGE 65 +				
Sex										
Male	49.88	10,342 (43.17)	1620 (6.76)			45.21	5386 (34.21)	1731 (11)		
Female	50.12	10,471 (43.71)	1524 (6.36)	1.07 (0.92–1.24)	0.32	54.79	6520 (41.42)	2105 (13.37)	0.99 (0.88–1.12)	0.94
Physiological conditions										
Has Asthma										
No	92.07	19,413 (81.04)	2642 (11.03)	2.01 (1.42–2.85)	0.000	92.91	11,233 (71.3)	3398 (21.5)	1.76 (0.39–2.24)	0.000
Yes	7.82	1386 (5.79)	487 (2.03)			7	669 (4.26)	429 (2.7)		
Has Arthritis										
No	81.57	17,760 (74.1)	1795 (7.50)	3.6 (2.91–4.51)	0.000	42.8	7423 (47.1)	1528 (9.7)	2.15 (1.85–2.50)	0.000
Yes	18.25	3038 (12.64)	1329 (5.55)			56.8	4447 (28.2)	2289 (14.5)		
Has Hypertension										
No	79.21	17,097 (71.37)	1898 (7.93)	2.4 (1.81–3.33)	0.000	52.1	6676 (42.4)	1536 (9.7)	1.70 (1.47–1.96)	0.000
Yes	20.56	3682 (15.37)	1224 (5.11)			47.6	5210 (3.1)	2285 (14.5)		
Has Diabetes										
No	92.53	19,744 (82.42)	2424 (10.12)	5.06 (4.12–6.23)	0.000	82.2	10,248 (65.1)	2700 (17.1)	2.49 (2.13–2.91)	0.000
Yes	7.42	1061 (4.43)	715 (2.99)			17.7	1653 (10.5)	1132 (7.19)		
Has Heart disease										
No	95.74	20,297 (84.73)	2639 (11.02)	5.19 (3.70–7.28)	0.000	80.5	10,145 (64.4)	2531 (16)	2.31 (1.89–2.83)	0.000
Yes	4.13	499 (2.08)	489 (2.04)			19.1	1730 (10.9)	1279 (8.1)		
Has Cancer										
No	98.01	20,546 (85.77)	2934 (12.25)	3.18 (1.90–5.31)	0.000	93.2	11,287 (71.7)	3386 (21.5)	1.75 (1.32–2.30)	0.000
Yes	1.90	256 (1.07)	199 (0.83)			6.63	608 (3.8)	435 (2.7)		
Has Anxiety disorder										
No	94.26	19,969 (83.36)	2612 (10.91)	3.4 (2.59–4.64)	0.000	95.6	11,559 (73.4)	3504 (22.2)	2.01 (1.35–2.99)	0.001
Yes	5.61	828 (3.46)	516 (2.15)			4.12	330 (2.1)	318 (2.02)		
Social determinants										
Marital Status										
Married	56.03	4424 (48)	741 (8)	1.00		50.64	1576 (38.95)	463 (11.4)	1.00	
Common-Law	7.99	628 (6.8)	101 (1.1)	1.03 (0.81–1.30)	0.79	2.60	66 (1.65)	38 (0.96)	0.89 (0.61–1.30)	0.55
Widow/Sep/Div	19.24	1542 (14)	232 (2.5)	0.53 (0.45–0.64)	0.28	41	1261 (31.17)	398 (6.86)	0.73 (0.64–0.83)	0.49
Single	16.34	1294 (14.05)	212 (2.31)	0.64 (0.53–0.77)	0.32	5.59	173 (4.28)	53 (1.32)	0.63 (0.16–0.64)	0.36
Level of Education										
Less than Secondary	11.2	1970 (11.2)	731 (3.06)	1.00		32.8	1443 (21.8)	1725 (10.9)	1.00	
Secondary Grad	17.50	3611 (15.08)	585 (2.45)	2.28 (1.80–2.90)	0.00	15.3	1944 (12.3)	466 (2.9)	2.08 (1.68–2.59)	0.00
Other post secondary	5.61	1121 (4.6)	222 (0.93)	1.87 (1.36–2.56)	0.00	5.76	709 (4.5)	199 (1.2)	1.78 (1.32–2.38)	0.00
Post secondary grad	62.59	13,514 (56.14)	1488 (6.21)	3.37 (2.76–4.10)	0.00	41.7	5314 (33.7)	1256 (7.9)	2.11 (1.84–2.43)	0.00
Total household Income										
<$20,000	6.33	920 (3.8)	596 (2.49)	1.00		11.4	1194 (7.5)	602 (3.8)	1.00	
$20,000-$39,999	11.21	2143 (8.9)	536 (2.24)	2.59 (2.05–3.27)	0.00	26.4	2988 (18.9)	1180 (7.5)	1.27 (1.07–1.52)	0.006
$40,000-$59,999	13.93	2877 (12.01)	462 (1.93)	4.02 (3.04–5.33)	0.00	17.4	2192 (13.9)	562 (3.5)	1.96 (1.59–2.41)	0.00
$60,000-$79,999	14.32	3095 (12.92)	338 (1.41)	5.92 (4.42–7.93)	0.00	8.8	1157 (7.3)	236 (1.5)	2.47 (1.85–3.28)	0.00
$80,000 or more	39.32	8728 (36.4)	705 (2.95)	8.01 (6.19–10.3)	0.00	12.55	1641 (10.4)	331 (2.1)	2.49 (1.88–3.30)	0.00
Psychological determinants										
Perceived Life Stress										
Quite a bit stressful	23.12	4578 (19.11)	965 (4.03)	1.00		9.29	868 (5.5)	692 (4.4)	1.00	
Not at all stressful	10	2231 (9.31)	180 (0.75)	2.61 (2.03–3.35)	0.00	25.9	3435 (21.8)	651 (4.1)	4.20 (3.39–5.22)	0.00
Not very stressful	19.7	4300 (17.95)	433 (1.81)	2.09 (1.65–2.64)	0.00	30.3	3867 (24.5)	902 (5.7)	3.41 (2.79–4.18)	0.00
A bit stressful	41.9	8863 (37)	1182 (4.94)	1.58 (1.30–1.91)	0.00	31.3	3553 (22.5)	1353 (8.6)	2.09 (1.71–2.55)	0.00
Extremely stressful	4.85	796 (3.33)	364 (1.52)	0.46 (0.34–0.61)	0.00	2.02	117 (0.75)	201 (1.2)	0.46 (0.30–0.69)	0.00

## Results

In the 40–64 age group, 86.8% of all respondents reported good health and 13.3% reported less than good health. Among those who reported less than good health, 48.4% were female and 51.5% were male. In the 65 and above age group, 75.6% of all respondents reported good health and 24.3% reported less than good health. 54.7 of those who reported less than good health were female and 45.2% were male.

Significant associations between self-reported health status and select social determinants, psychological determinants and physiological conditions are presented for both the younger age group (40–64 years) and the older age group (65+ years). In general, self-reported health status was inversely correlated with age. In the older age group (65+ years), the absence of chronic health conditions was strongly predictive of good self-reported health. The absence of cancer (OR=3.18), heart disease (OR=2.85) and diabetes (OR=2.31) demonstrated the strongest associations with self-reported “*good health*” in this age category.

In the younger age group (40–64 years), the absence of chronic health conditions was even more strongly predictive of self-reported “*good health*”. As with older individuals, the absence of cancer (OR=4.98), heart disease (OR=4.71) and diabetes (OR=4.14) were most strongly associated with perceived “*good health*” (see Table 2).

**Table 2:** Odds ratios of variables associated with good self-perceived health for Canadians aged 40–46 and 65+ using multivariate logistic regressions

Variables	AGE 40–64		AGE 65+	
Good Health (1) vs. Less than good (0)	OR	*P*	OR	*P*
Sex				
Female	1.27 (1.08–1.50)	0.001	1.37 (1.12–1.66)	0.001
Physiological conditions				
Has Asthma				
No	1.43 (1.16–1.76)	0.001	1.81 (1.56–2.52)	0.000
Has Arthritis				
No	2.93 (2.42–3.55)	0.000	1.99 (1.73–2.28)	0.000
Has Hypertension				
No	1.59 (1.32–1.91)	0.000	1.38 (1.20–1.59)	0.000
Has Diabetes				
No	4.14 (3.19–5.35)	0.000	2.31 (1.83–2.61)	0.000
Has Heart disease				
No	4.71 (3.22–6.28)	0.000	2.85 (2.10–2.97)	0.000
Has Cancer				
No	4.98 (3.5–7.1)	0.000	3.18 (1.81–3.30)	0.000
Has Anxiety disorder				
No	2.8 (2.19–3.65)	0.000	2.02 (1.50–2.71)	0.000
Social determinants				
Level of Education				
Less than secondary	1		1	
Secondary Grad	1.45 (1.08–1.95)	0.01	1.84 (1.47–2.30)	0.000
Other post secondary	1.37 (0.98–1.90)	0.05	1.72 (1.24–2.37)	0.001
Post secondary grad	2.07 (1.65–2.60)	0.00	1.79 (1.51–2.11)	0.000
Total household Income				
<$20,000	1		1	
$20,000–$39,999	2.26 (1.69–3.03)	0.000	1.17 (0.95–1.44)	0.13
$40,000–$59,999	2.73 (1.98–3.76)	0.000	1.52 (1.18–1.95)	0.001
$60,000–$79,999	3.97 (2.83–5.55)	0.000	1.78 (1.28–2.46)	0.001
$80,000 or more	4.65 (3.23–6.05)	0.000	1.94 (1.37–2.67)	0.000
Psychological determinants				
Perceived Life Stress				
Quite a bit	1		1	
Not at all	2.78 (2.25–4.05)	0.000	3.83 (3.02–4.92)	0.000
Not stressful	2.11 (1.59–2.82)	0.000	2.97 (2.35–3.74)	0.000
A bit stressful	1.51 (1.21–1.89)	0.000	2.04 (1.63–2.56)	0.000
Extremely stressful	0.64 (0.47–0.86)	0.004	0.51 (0.31–0.83)	0.007

In the 65+ age group, individuals in the highest income bracket ($80,000+) were 1.94 times more likely to report “*good health*” than those individuals in the lowest income bracket ($0–20,000). This association was much stronger (OR=4.65) in the younger age group; this is an important finding because it may be an indication that the protections available to seniors, and not available to the younger age group, are somewhat effective in protecting them.

The relationship between the level of education and self-reported health was also consistent with our expectations. More education seems to be associated with reporting better health. In the 65 and older age group, individuals who graduated from secondary school were more likely to report “*good health*” than those with less than secondary education (OR=1.84). Among participants in the younger age group, secondary school graduates were also more likely to report “*good health*” compared to individuals with less than secondary education (OR = 1.45). Table 2 summarizes the odds ratio estimates for select social factors.

Individuals in the older age group (65+) who described their life as “not at all stressful” were 3.83 times more likely to report “*good health*” than those who described their life as “quite a bit stressful”; this association was also strong in the 40–64 age group (OR=2.78).

## Discussion

The first objective of this study was to identify determinants of self-perceived health in Canada in two age groups (40–64 years and 65 and older years). Significant determinants of self-reported health found among both age groups include the presence of chronic conditions, household income and perceived life stress.

In both age groups, the presence of chronic health conditions such as cancer, heart disease and diabetes resulted in statistically lower odds of reporting “*good health*”. Comparing the two age groups, individuals in the younger age group (40–64) demonstrated lower odds of self-perceived “*good health*” for each chronic condition compared to their counterparts in the older age group (65+) with similar conditions. Prior research has demonstrated that chronic disease and comorbid conditions significantly contribute to poorer self-perceived health [26, 27]. This is because self-perceived health is not only affected by medical facts and biological health but also how illness affects day-to-day living [28–30]. It is widely accepted that self-perceived health is a multidimensional construct determined not only by the absence of health problems but also functional, coping and wellbeing factors [28, 31, 32]. Additionally, self-perceived health may be influenced by family history and a personal estimate of longevity [6]. Idler and Benyamini [33] proposed that self-perceived health was a dynamic measure, accounting not only for present levels of health but a judgment of the trajectory of future health. These findings may partially explain why participants in both age groups with chronic diseases were less likely to report “*good health*”: that is, because chronic diseases impede on functional, coping and wellbeing factors, individuals may recognize these shortcomings in the way they perceive their own health. The literature also provides some insight into why the younger age group (40–64) with chronic conditions may be less likely to report “*good health*” compared to participants in the older age group (65 and older). One such insight is that experiences of chronic conditions among the younger age group (40–64) may result in a poorer judgement of the trajectory of future health as these experiences will likely last for a larger portion of an individual’s life course.

We found that across both age groups, higher levels of stress were associated with lower odds of self-perceived “*good health*”. However, the older group was even less likely to report “*good health*” in the higher stress categories. This is unsurprising given that psychosocial factors, notably stress, have been historically linked to poorer health perceptions [34]. The mechanism by which stress is linked to poorer health perceptions may relate to the ways stress influences physical health. Distress, or feeling stressed, may increase the risk of coronary heart disease, [35, 36] may play a role in the development of metabolic disorders, [37, 38] and can reduce the body’s immune response [39]. Distress is also associated with higher all-cause mortality and exhibits a dose–response relationship when adjusting for comorbid conditions, behaviours and socioeconomic status (SES) [40]. Older adults in the 65+ age group who experienced higher stress levels were more likely to report poorer health than their highly stressed counterparts in the younger age group (45–64). Choi and Jun [41] argue that older adults experience a number of unfamiliar stressors unique to ageing, including loneliness, having to depend on others for support, and caregiving for a spouse or relative, which could contribute to higher odds of reporting “*poor health*”.

As for the relationship between income and self-perceived health, there is a large body of research documenting the relationship between SES and self-perceived health, whether using measures of income, occupation or education as a SES metric [42, 43]. Our results demonstrated that respondents in both age groups in the lowest quintile of household income (<$20,000) were more likely to report poorer health. This relationship could be explained either by health selection or social causation. Health selection proposes that the relationship between lower SES strata and poorer self-perceived health is due to the detrimental effect that poor health has on educational and occupational attainment, thereby precipitating a downward spiral in SES [44]. This model posits that respondents in both age groups in the lowest quintile of household income (<$20,000) are also those already in poor health, and this is reflected in their reports of self-perceived health. In contrast, social causation models explain that persons in lower SES strata are subject to conditions such as poor housing, poor nutrition, inadequate education and access to medical services that trigger poorer health outcomes [45]. According to this model, the conditions experienced by respondents in both age groups in the lowest quintile of household income (<$20,000) are associated with poor health, and so living in these conditions negatively impacts self-perceived health. Despite contrasting theories as to the directionality of the relationship, our results are complimentary to the widely accepted relationship between income and self-perceived health presented in the literature.

Our findings are likely interrelated and reflect the complexities of health, perceptions of health and its relationship with social, psychological and physiological determinants. For example, another mechanism by which income may affect self-perceived health is through the relationship between income security and distress. Orpana et al. [46] demonstrated evidence that the measure of income in the form of financial stability may influence level of distress and, therefore, alter self-perceived health. This mechanism showcases interrelations between self-perceived health and two distinct categories of determinants: social and psychological. This level of analysis and assessment will likely be necessary moving forward when addressing self-perceived health disparities.

Our findings extend the existing literature by focusing specifically on the Canadian population and demonstrate that among middle-aged and elderly Canadians, the presence of chronic conditions, lower household income and psychological distress result in higher odds of lower self-perceived health status.

### Limitations

The 2010 Canadian Community Health Survey (CCHS) is a cross-sectional study conducted across all 10 Canadian provinces and three territories [25]. Its cross-sectional design only allows for identification of associations, but not causation. Furthermore, the CCHS is limited as it directly excludes certain population groups such as persons living on reserves or other Aboriginal settlements, full-time members of the Canadian Forces, the institutionalized population and inhabitants within the Québec health regions: Région de Nunavik and Région des Terres-Cries-de-la-Baie-James. Indirectly, the CCHS survey excludes Canadians without stable housing and/or a registered home telephone number. Finally, since participation is voluntary, the data collected may reflect a certain level of volunteer bias.

Self-perceived health, although proven a good predictor of health status and mortality, [6–9] is still assumed to be an inferior indicator of population health compared to objective health measures [47]. Some limitations of the self-perceived health measure include reporting bias and difficulties interpreting this measure across varying age ranges and cultural groups. Although there are limitations, a recent study by Schnittker and Bacak [48] suggests that the self-perceived health measure is becoming increasingly more predictive of objective health and mortality due to the widespread availability of health information in developed countries. Finally, we were not able to include some variables, such as “social ties”, because they were not available in the dataset.

## Conclusions and policy implications

Findings from longitudinal analyses have demonstrated the power of the self-perceived health measure in predicting the incidence of chronic disease, [7–9] recovery from illness, [49] functional decline, [50] use of medical services, [14, 15] and even mortality [6–9].

The findings of this study indicate that chronic conditions, income and stress all have significant influences on the self-perceived health status of middle-aged and elderly Canadians. Since self-perceived health status within a population can affect medical service utilization and mortality rates, it is critical that policymakers identify and employ measures to alleviate the negative effects of these factors on individuals’ self-perceived health status. The results also highlight the importance of social determinants of health. It is clear that in spite of having a universal adequate health insurance system, inequities associated with income persist in Canada.

Lorig et al. [51] found that a chronic disease self-management program over 2 years in persons 40 years and older reduced medical service utilization, improved health distress and improved patient self-efficacy. Weighing the costs and benefits of such an intervention, Lorig et al demonstrated that their intervention prevented approximately 2.5 visits for medical services (emergency room visits & physician outpatient visits) per participant and reduced hospitalization stays by 0.5 days per participant. This reduced use of medical services saved roughly $590 in healthcare costs per participant while the intervention itself cost between $70–200 per participant. The former is just one example of a targeted program or policy, which could help improve health among a specific population group while concurrently reducing health care costs.

Other policies to address income stability, financial security and distress among older Canadians could also serve to improve the health status of this population group while simultaneously reducing health care costs. When looking at financial security, although Canada has one of the lowest elderly poverty rates in the developed world, elderly women living alone (widowed/divorced/never married) tend to be at the highest risk of being impoverished due to pension allowances tied to previous employment [52]. Inadequate financial security in later life can severely limit access to resources, which may improve health and quality of life, such as transportation, social participation and adequate nutrition. Also, studies have shown a link between financial insecurity among elderly and increased levels of distress [53]. Policies concerning elderly poverty should take this into consideration and poverty among elderly women/men living alone could be reduced through increased government transfers to this population. It is important for policymakers to incorporate and address the social determinants of health in order to facilitate healthy public policy.

In conclusion, the presence of chronic conditions, distress and lower household income negatively impacts the odds of reporting “good” health among middle-aged and elderly Canadians. Targeted policies addressing these factors are likely to lead to significant reductions in identified health inequities.
